# Genome-wide association scan for QTL and their positional candidate genes associated with internal organ traits in chickens

**DOI:** 10.1186/s12864-019-6040-3

**Published:** 2019-08-22

**Authors:** Gabriel Costa Monteiro Moreira, Mayara Salvian, Clarissa Boschiero, Aline Silva Mello Cesar, James M. Reecy, Thaís Fernanda Godoy, Mônica Corrêa Ledur, Dorian Garrick, Gerson Barreto Mourão, Luiz L. Coutinho

**Affiliations:** 1 University of São Paulo (USP), Luiz de Queiroz College of Agriculture (ESALQ), Piracicaba, São Paulo, Brazil; 2 Department of Animal Science, Iowa State University (ISU), Ames, Iowa USA; 3 Embrapa Suínos e Aves, Concórdia, Santa Catarina Brazil; 4 School of Agriculture, Massey University, Ruakura, Hamilton, New Zealand

**Keywords:** GWAS, Heart, Liver, Gizzard, Lungs, Intestine, Genomic heritability, Deleterious mutations, Metabolic disorders

## Abstract

**Background:**

Poultry breeding programs have been focused on improvement of growth and carcass traits, however, this has resulted in correlated changes in internal organ weights and increased incidence of metabolic disorders. These disorders can affect feed efficiency or even cause death. We used a high density SNP array (600 K, Affymetrix) to estimate genomic heritability, perform genome-wide association analysis, and identify genomic regions and positional candidate genes (PCGs) associated with internal organ traits in an F2 chicken population. We integrated knowledge of haplotype blocks, selection signature regions and sequencing data to refine the list of PCGs.

**Results:**

Estimated genomic heritability for internal organ traits in chickens ranged from low (LUNGWT, 0.06) to high (GIZZWT, 0.45). A total of 20 unique 1 Mb windows identified on GGA1, 2, 4, 7, 12, 15, 18, 19, 21, 27 and 28 were significantly associated with intestine length, and weights or percentages of liver, gizzard or lungs. Within these windows, 14 PCGs were identified based on their biological functions: *TNFSF11*, *GTF2F2*, *SPERT*, *KCTD4*, *HTR2A*, *RB1*, *PCDH7*, *LCORL*, *LDB2*, *NR4A2*, *GPD2*, *PTPN11*, *ITGB4* and *SLC6A4*. From those genes, two were located within haplotype blocks and three overlapped with selection signature regions. A total of 13,748 annotated sequence SNPs were in the 14 PCGs, including 156 SNPs in coding regions (124 synonymous, 26 non-synonymous, and 6 splice variants). Seven deleterious SNPs were identified in *TNFSF11*, *NR4A2* or *ITGB4* genes.

**Conclusions:**

The results from this study provide novel insights to understand the genetic architecture of internal organ traits in chickens. The QTL detection performed using a high density SNP array covered the whole genome allowing the discovery of novel QTL associated with organ traits. We identified PCGs within the QTL involved in biological processes that may regulate internal organ growth and development. Potential functional genetic variations were identified generating crucial information that, after validation, might be used in poultry breeding programs to reduce the occurrence of metabolic disorders.

**Electronic supplementary material:**

The online version of this article (10.1186/s12864-019-6040-3) contains supplementary material, which is available to authorized users.

## Background

Poultry breeding programs have for many generations been selecting animals mainly based on feed efficiency, growth, performance and carcass traits, achieving significant improvements in broiler chicken production [1–4]. However, muscle development has been disproportionate to development of internal organs, increasing the susceptibility of improved broiler chickens to metabolic disorders, and death [5–7].

Although the weights of internal organs have not been direct targets for selection in poultry breeding programs, feed intake (commonly targetted for selection) has exhibited positive genetic correlations with heart weight (0.77), liver weight (0.73) and intestine weight (0.92) in a meat-type chicken population [8]. Thus, the detection of genomic regions and/or potentially causative mutations associated with internal organ weight traits may provide important information to poultry breeding programs, facilitating selection of chickens with proportional development of internal organs, improved feed efficiency and reduced susceptibility to metabolic disorders.

The pulmonary and cardiac capacities of commercial broilers are similar to those observed in non-selected chickens [9], despite their larger body size, such that the heart and lungs can be overloaded [10, 11]. This increases the occurrence of ascites, a metabolic disorder characterized by accumulation of fluid in the abdominal cavity of birds [10, 11], hypertension syndrome (PHS) [12, 13] or even heart failure and sudden death syndrome (SDS) [14–17]. Moreover, changes in the weight of the gizzard can influence nutrient availability in the small intestine, and intestine length is associated with feed efficiency and growth in chickens [18, 19]. Long-term intense selection for carcass traits can also increase the occurrence of fatty liver syndrome [20]. Thus, the discovery of genomic regions and positional candidate genes (PCGs) for intestine length or weights and percentages of heart, liver, gizzard or lungs, is a first step for understanding their genetic architecture.

Only a few QTL mapping studies for internal organs have been performed in chickens [5, 6, 21–26] and the genetic architecture of these traits is still unclear. Recently, a GWAS study in a layer Chinese F2 population using a high density SNP array (600 K Axiom Chicken Genotyping Array) [27] used the exact mixed model approach in the GEMMA package [28] and identified 23 highly significant SNPs associated with the weights of heart, liver, proventriculus and gizzard. A total of 96 QTL mapped for the same internal organs traits evaluated in this study have been curated in the Chicken QTL database – release 35 [29] and, from those, only 16 are well annotated (exhibit start and end position properly updated to *Gallus_gallus-5.0* genome assembly): four for gizzard weight (QTL #35268, QTL #96657, QTL #96660, QTL #96664), one for gizzard percentage (QTL #35262), two for heart weight (QTL #137389, QTL #56402), one for heart percentage (QTL #35263), one for liver weight (QTL #56401), one for lung weight (QTL #137387), and six for intestine length (QTL #96673, QTL #56393, QTL #56394, QTL #56398, QTL #56403, QTL #96632).

The use of a high density SNP panel covering the whole genome combined with genomic prediction methodology can provide more accurate QTL detection and facilitate the identification of positional candidate genes (PCGs). The main goal of this study was to estimate genomic heritability, and detect QTL and PCGs for internal organs weights and percentages in chickens. Knowledge of haplotype blocks, selection signature regions and the use of sequencing data were integrated to refine the list of PCGs.

## Results

### Descriptive statistics and genomic heritability

The number of chickens analyzed for each internal organ trait, along with the averages, standard deviation, variance components (genetic, residual and total variances) and genomic heritability are in Table 1. The estimated genomic heritability ranged from low (0.06) to high (0.45).

**Table 1:** Descriptive statistics, variance components and genomic heritability for internal organ traits in the Embrapa F_2_ Chicken Resource Population

Trait	N	Average ± SD^1^	Residual variance	Genetic variance	Total variance	Genomic heritability^2^
HWT	480	6.389 ± 1.694	1.149	0.131	1.279	0.10
HEARTP	480	0.658 ± 0.131	0.013	0.002	0.014	0.12
LIVWT	480	26.139 ± 5.308	7.481	0.565	8.046	0.07
LIVP	480	2.695 ± 0.326	0.068	0.014	0.082	0.17
GIZZWT	480	23.830 ± 4.533	6.398	5.309	11.707	0.45
GIZZP	480	2.476 ± 0.399	0.074	0.060	0.134	0.44
LUNGWT	479	8.222 ± 2.246	2.606	0.163	2.769	0.06
LUNGP	479	0.817 ± 0.165	0.027	0.003	0.030	0.10
INTES	479	153.056 ± 14.793	88.588	61.656	150.244	0.41

*HWT*: heart weight; *HEARTP*: heart percentage; *LIVWT*: liver weight; *LIVP*: liver percentage; *GIZZWT*: gizzard weight; *GIZZP*: gizzard percentage; *LUNGWT*: lung weight; *LUNGP*: lung percentage; *INTES*: intestine length (cm). All the weights are expressed in grams

^1^Standard deviation of the mean

^2^Genomic heritability estimated with Bayes B model

### Genome-wide association study (GWAS)

**Fig. 1:**
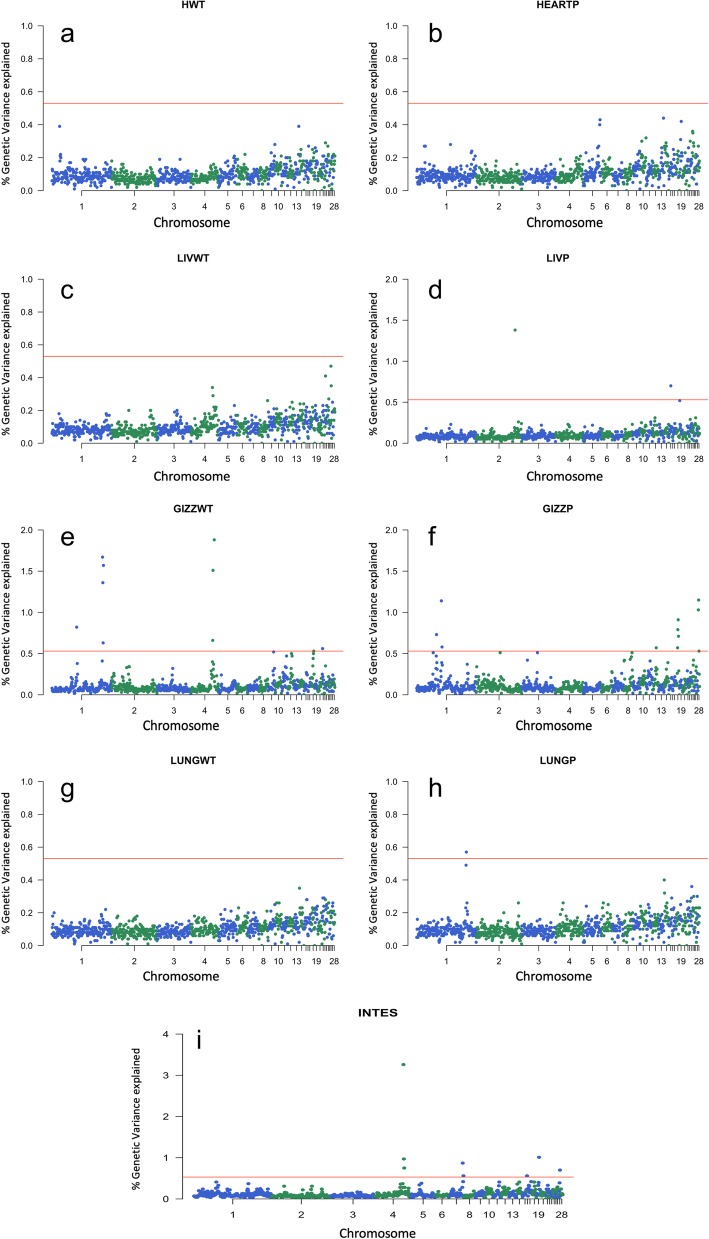
Manhattan plots of the posterior means of the percentage of genetic variance explained by each 1 Mb SNP window across the 28 autosomal chromosomes for heart weight (**a**), heart weight as a percentage of body weight (**b**), liver weight (**c**), liver weight as a percentage (**d**), gizzard weight (**e**), gizzard weight as a percentage (**f**), lung weight (**g**), lung weight as a percentage (**h**), and intestine length (**i**). The X-axis represents the chromosomes, and Y-axis shows the proportion of genetic variance explained by each window from Bayes B analysis. Red lines indicate the threshold to deem significant SNP windows

We estimated the proportion of the genetic variance explained by each one of the 943 non-overlapping 1 Mb windows for all the traits analyzed (Additional file 1). The Manhattan plots of the posterior means of the proportion of genetic variance explained by each SNP window across the 28 autosomal chromosomes for internal organ traits are in Fig. 1.

The characterization of the significant genomic windows is in Table 2. A total of 28 significant 1 Mb windows (with different positions) were identified on GGA1, 2, 4, 7, 12, 15, 18, 19, 21, 27, and 28 for LIVP, GIZZWT, GIZZP, LUNGP and INTES. The posterior probability of association (PPA) [30] ranged from 0.38 to 0.8 for each significant region. The percentage of the variance explained by the window ranged from 0.53 to 3.26.

**Table 2:** Characterization of 1 Mb significant genomic windows for internal organ traits in the Embrapa F_2_ Chicken Resource Population

Trait	GGA_Mb^1^	SNP window (first – last position)^1^	Number of SNP/ window	Proportion of genetic variance explained by the window	PPA^2^
LIVP	2_127	rs317047746 - rs317596831	281	1.38	0.38
	15_6	rs318148607 - rs313939736	511	0.70	0.47
GIZZWT	1_81	rs316112843 - rs14849734	355	0.82	0.50
	1_166	rs15488636 - rs13551715	418	1.67	0.62
	1_167	rs14913877 - rs13552288	358	1.36	0.52
	1_168	rs318211853 - rs15497155	318	0.63	0.47
	1_169	rs315581943 - rs13972577	346	1.57	0.53
	4_71	rs317228804 - rs316204639	330	0.66	0.42
	4_72	rs16433889 - rs316997412	281	1.51	0.48
	4_76	rs15618974 - rs314892344	308	1.88	0.47
	18_7	rs312913684 - rs312861933	425	0.53	0.53
	21_1	rs16177395 - rs318084617	877	0.56	0.73
GIZZP	1_65	rs317208413 - rs15299359	415	0.73	0.51
	1_81	rs316112843 - rs14849734	355	1.14	0.56
	1_83	rs317965743 - rs15328274	382	0.58	0.51
	12_12	rs316348330 - rs315497876	472	0.57	0.55
	18_4	rs315188853 - rs315807623	643	0.57	0.65
	18_5	rs312939477 - rs317197824	790	0.79	0.72
	18_6	rs313941377 - rs317640358	791	0.91	0.72
	18_7	rs312913684 - rs312861933	425	0.71	0.54
	28_0	rs313774457 - rs312701176	829	1.03	0.80
	28_1	rs14305593 - rs314996946	560	1.15	0.65
	28_2	rs313767061 - rs317469562	659	0.53	0.63
LUNGP	1_163	rs314645027 - rs15485042	293	0.57	0.42
INTES	4_71	rs317228804 - rs316204639	330	3.26	0.64
	4_72	rs16433889 - rs316997412	281	0.97	0.42
	4_73	rs317373306 - rs15617120	284	0.75	0.36
	7_34	rs316467562 - rs312928601	411	0.87	0.56
	7_36	rs316261866 - rs315360554	257	0.56	0.40
	15_11	rs317306003 - rs314017312	473	0.56	0.59
	19_6	rs313367474 - rs314881460	602	1.01	0.72
	27_3	rs14302748 - rs312772391	820	0.70	0.76

*LIVP*: liver percentage; *GIZZWT*: gizzard weight; *GIZZP*: gizzard percentage; *LUNGP*: lung percentage; *INTES*: intestine length

^1^Map position based on *Gallus_gallus*-5.0 assembly (NCBI)

^2^Posterior probability of association (PPA) as described by Onteru et al. [30]

Adjacent genomic windows associated with the same trait were considered as the same QTL and their respective proportions of the variance explained were summed. For GIZZWT, two QTL were identified: one on GGA1 (rs15488636- rs13972577) explaining 5.23% of the genetic variance and one on GGA4 (rs317228804 - rs316997412) explaining 2.17% of the genetic variance. For GIZZP two QTL were identified: one on GGA18 (rs315188853 - rs312861933) explaining 2.98% of the genetic variance and one on GGA28 (rs313774457- rs317469562) explaining 2.71% of the genetic variance. For INTES, one QTL was identified on GGA4 (rs317228804 - rs15617120) explaining 4.98% of the genetic variance. Therefore, considering the adjacent windows associated with the same trait, as the same QTL, a total of 20 QTL were detected herein.

### Overlap with previously reported QTL

Sixteen QTL identified in this study have previously been annotated/converted in *Gallus_gallus*-5.0 assembly, available in the Chicken QTL database – release 35 [29]. Three detected QTL overlapped with known QTL mapped for internal organ traits (Table 3) in different populations; one detected in a Brazilian paternal broiler population through SNP association analysis (QTL #137389) [23], and two mapped in a meat-type chicken population derived from a F2 cross through linkage analyses by interval mapping method (QTL #96657, QTL #96660) [21].

**Table 3:** Overlap between the detected QTL and published QTL for internal organ traits in chickens

GGA (Mb)	Genomic position^1^	Associated trait	Known QTL (associated trait)^2^
1 (166–170)	rs15488636 – rs13972577	GIZZWT	QTL #137389 (HWT)
19 (6)	rs313367474 – rs314881460	INTES	QTL #96657 (GIZZWT)
21 (1)	rs16177395 – rs318084617	GIZZWT	QTL #96660 (GIZZWT)

*HWT*: heart weight; *GIZZWT*: gizzard weight; *INTES*: intestine length

^1^Genomic positions based on *Gallus_gallus*-5.0 assembly (NCBI)

^2^Chicken QTLdb ID numbers database – release 35 [29]

The QTL associated with GIZZWT on GGA1 (rs15488636 – rs13972577) and on GGA4 (rs317228804 - rs316204639) overlapped with those previously reported on GGA1 (165.93–172.12 Mb), and on GGA4 (71.30–77.19 Mb) for the same trait in a GWAS performed using a high density SNP array (600 K, Affymetrix) with layer chickens from a Chinese F2 population [31].

Four of the 20 QTL overlapped with QTL previously reported for the same population for fatness traits using the same methods applied in this study [32] (Table 4).

**Table 4:** – Overlaps between the 20 QTL detected herein, and QTL previously mapped for fatness traits using the same chicken population (Embrapa F_2_ Chicken Resource Population)

GGA_Mb	Genomic window	Associated trait	Fatness associated trait [32]
	(first and last SNP)		
1 (166–170)	rs15488636 – rs13972577	GIZZWT	CFC
7_36	rs316261866 - rs315360554	INTES	CFC, CFCDM
27_3	rs14302748 - rs312772391	INTES	ABF
28_0	rs313774457 - rs312701176	GIZZP	ABFP

*ABF*: abdominal fat weight in grams; *ABFP*: abdominal fat percentage; *CFC*: carcass fat content in grams; *CFCDM*: carcass fat content on a dry matter basis

^1^Map position based on *Gallus_gallus*-5.0, NCBI assembly

### Positional candidate genes

A total of 756 genes have been annotated in the regions defined by the 20 QTL (Additional file 2). Among these, 14 PCG (Table 5) were identified as promising candidates based on their Gene Ontology (GO) terms and information available in the literature. The characterization of selection signature regions overlapping with the associated genomic windows and the characterization of haplotype blocks harboring the SNPs with the highest model frequency in each window are available in Additional files 3 and 4, respectively.

**Table 5:** Genomic windows associated with internal organ traits that harbor PCGs

GGA (Mb)	Trait associated	PCG^1^	Ensembl gene ID^2^
1 (166)	GIZZWT	*TNFSF11*	ENSGALG00000026163
1 (167)	GIZZWT	*GTF2F2*	ENSGALG00000016974
		*SPERT*	ENSGALG00000016981
		*KCTD4*	ENSGALG00000016975
1 (168)	GIZZWT	*HTR2A*	ENSGALG00000016992
		*RB1*	ENSGALG00000016997
4 (72)	INTES, GIZZWT	*PCDH7* ^*3,4*^	ENSGALG00000033883
4 (76)	GIZZWT	*LCORL* ^*4*^	ENSGALG00000014421
		*LDB2*	ENSGALG00000014485
7 (36)	INTES	*NR4A2* ^*3*^	ENSGALG00000012538
		*GPD2* ^*3*^	ENSGALG00000012543
15 (6)	LIVP	*PTPN11*	ENSGALG00000004821
18 (4)	GIZZP	*ITGB4*	ENSGALG00000002389
19 (6)	INTES	*SLC6A4*	ENSGALG00000004246

^1^Positional candidate genes

^2^Ensembl gene ID based on Galgal5 (*Ensembl Genes 93 Database*)

^3^Indicates that the positional candidate gene was annotated within a selection signature region [33]

^4^Indicates that this positional candidate gene was annotated within a haplotype block that harbor the SNP with the highest model frequency

### SNPs located in positional candidate genes

A total of 13,748 SNPs were annotated in the 14 PCGs. From this total, approximately 2% exhibited multiple annotations, being classified in different annotation categories as reported by Moreira et al. [34]. The number of SNPs annotated and their respective locations in each PCG is in Table 6.

**Table 6:** Functional annotation of SNP identified in the 14 positional candidate genes, from sequencing data of 28 parental chickens of the Embrapa F_2_ Chicken Resource Population

Genetic variants annotation	Positional candidate genes													
	***PTPN11***	***RB1***	***TNFSF11***	***ITGB4***	***SLC6A4***	***GTF2F2***	***SPERT***	***KCTD4***	***HTR2A***	***PCDH7***	***LCORL***	***LDB2***	***NR4A2***	***GPD2***
3′ UTR^1^	–	20	–	–	–	10	–	6	–	–	5	3	14	–
5′ UTR^2^	1	2	–	–	–	1	–	–	–	–	–	–	1	–
Downstream	57	94	104	127	70	129	54	80	60	16	127	80	83	88
Intron	290	1431	332	436	150	1849	13	–	404	1789	811	3138	14	490
Non-synonymous	–	2	3	9	4	–	–	–	–	1	–	–	7	–
Splice region (synonymous)	–	–	–	3	2	–	–	–	–	–	–	–	1	–
Splice region (intron)	2	3	–	8	–	–	–	–	–	–	–	–	–	–
Synonymous	5	15	4	49	11	2	2	1	3	7	–	1	16	8
Upstream	97	101	90	296	89	90	66	106	64	12	52	42	60	35
***Total***	**452**	**1668**	**533**	**928**	**326**	**2081**	**135**	**193**	**531**	**1825**	**995**	**3264**	**196**	**621**

^1^Within 5 kb downstream of the 3 prime end of a transcript

^2^Within 5 kb upstream of the 5 prime end of a transcript

A total of 156 SNPs were annotated in coding regions (124 synonymous, 26 non-synonymous and six splice site variants) and for each of those, the SIFT score was used to predict deleterious mutations. Seven predicted deleterious SNPs were identified: one in the *TNFSF11* gene, three in the *NR4A2* gene, and three in the *ITGB4* gene (Table 7).

**Table 7:** Characterization of the seven predicted deleterious SNPs annotated in PCGs for internal organ traits in chickens

Gene Symbol	SNP ID	GGA	Position^1^	Amino acid change	SIFT score^2^
*TNFSF11*	rs312442403	1	166,510,185	Arg/Gln	Deleterious low confidence^3^ (0.03)
*NR4A2*	g.36224286 > C/T	7	36,224,286	Val/Met	Deleterious (0)
	g.36225242 > G/T		36,225,242	Arg/Ser	Deleterious (0)
	g.36225278 > C/T		36,225,278	Val/Met	Deleterious (0.01)
*ITGB4*	rs313279811	18	4,785,491	His/Tyr	Deleterious (0.01)
	rs316340790		4,787,392	Asp/Asn	Deleterious (0.04)
	rs732847450		4,790,177	Met/Ile	Deleterious (0.03)

^1^Position based on *Gallus_gallus* 5.0 assembly

^2^SIFT (Sorting Intolerant From Tolerant) score

^3^Deleterious low confidence: little sequence diversity in this position affecting the substitution model, and consequently the averages of conservation value [35]

## Discussion

### Descriptive statistics and genomic heritability

The means and standard deviations observed herein for HWT and LIVWT are in agreement with the values observed in a previous GWAS study for organ traits performed by Dou et al. [31], using an F2 population derived from reciprocal crosses between a commercial broiler line and an indigenous strain [31], but differ for GIZZWT. The means and standard deviations of the internal organ weights and percentages reported herein differ from those reported by Venturini et al. [36] and Gaya et al. [8], however, they used meat-type chickens from broiler lines selected over many generations for rapid growth and meat production [8, 36, 37]. Those differences are expected since the F2 population used herein was founded from reciprocal crosses between grandparental broiler and layer lines, that exhibit different phenotypic abilities [6, 38–40].

Our genomic heritability estimates ranged from low to high for the traits analyzed in the Embrapa F_2_ Chicken Resource population (Table 1). Previous studies reported heritability estimates using pedigree information for internal organ traits in meat-type chickens [8, 36, 41]. The heritability estimates for HWT, GIZZWT, and LIVWT reported in one study were 0.38, 0.39 and 0.25 [8], and in another 0.27, 0.44 and 0.33 [36]. Thus, the heritability estimates for GIZZWT reported in different populations (0.39 and 0.44) corroborates our finding (0.45). In addition to the expected differences in the heritabilities due to the methods used to estimate, it is important to consider that we used an F2 population obtained from reciprocal crossings between two distinct lines, thus, differences from the heritability estimated reported in meat-type populations would not be surprising.

A previous study using a larger dataset from the same chicken population comprising 2063 F_2_-TCTC population, reported pedigree-based heritability for HWT, LIVWT, GIZZWT, LUNGWT and INTES equal to 0.19 ± 0.04, 0.14 ± 0.05, 0.70 ± 0.05, 0.60 ± 0.05 and 0.04 ± 0.02, respectively [42]. The genomic heritability estimates for HWT and LIVWT were similar to the heritability reported herein and, the differences observed in GIZZWT, LUNGWT and INTES estimates could be due the reduced sample size used herein, which may not represent the genetic variance for these traits in the whole population.

In summary, our genomic heritability estimates ranged from low (0.06) to high (0.45) (Table 1). Based on the high genomic heritability estimated for GIZZWT, GIZZP and INTES, a response would be expected for selection focused on these traits.

### GWAS and QTL discovery

We detected 28 significant 1 Mb QTL associated with LIVP, GIZZWT, GIZZP, LUNGP or INTES explaining from 0.53 to 3.26% of the genetic variance, with the PPA ranging from 0.38 to 0.80 (Table 2).

Considering the adjacent associated QTL and their respective cumulative proportions of the variance explained, we detected QTL explaining 5.23% (on GGA1) and 2.17% (on GGA4) of the genetic variance for GIZZWT, 2.98% (on GGA18) and 2.71% (on GGA28) for GIZZP, and 4.98% (on GGA4) for INTES. A total of 20 QTL were detected and together, they explained 2.08, 11.19, 8.71, 0.57 and 8.68% of genetic variance for LIVP, GIZZWT, GIZZP, LUNGP and INTES, respectively. A low heritability can reduce the power to detect QTL [43], also explaining these greater number of QTL detected for the traits with high heritability estimates.

The low PPA values observed in our study can be due to the reduced sample size used herein, that can reduce the power to detect QTL [43]. However, the overlaps observed with known QTL available in the Chicken QTL database – release 35 [29], and also the detection of PCGs within the QTL associated with internal organ weights and percentage corroborate our findings.

Only 16 known QTL mapped for the internal organs traits evaluated in this study are well annotated (have genome position available) in the Chicken QTL database – release 35 [29]: four for GIZZWT, one for GIZZP, two for HWT, one for HEARTP, one for LIVWT, one for LUNGWT and six for INTES. Thus, not many overlaps between the 20 QTL detected herein with the 16 previously published QTL should be expected. Nevertheless, one QTL mapped for GIZZWT, and one mapped for INTES overlapped with known QTL mapped for internal organ traits (HWT and GIZZWT) in chickens, and one QTL for GIZZWT detected herein overlapped with one known QTL mapped for the same trait suggesting our findings are unlikely to represent false positive discoveries (Table 3). The QTL detected on GGA1 and GG4 explaining 5.23 and 2.17% of the genetic variance for GIZZWT, respectively, also overlapped with QTL detected in a GWAS performed using the high density SNP array (600 K, Affymetrix) and layer chickens from a Chinese F2 population [31], validating our findings.

We did not observe overlaps with the QTL detected by Moura et al. [5] in a mapping study performed with the same F2 population, using a different number of animals, low density of markers (approximately 127 microsatellite markers) and an interval mapping method. It is important to highlight that in the study by Moura et al. [5], only 11 significant QTL were detected, and from those, six showed similar effects across sexes [5] for HWT, GIZZWT, GIZZP and LIVP. Additionally, different QTL can be detected depending on the method and the density of markers used. In this context, in a study performed to identify QTL that control performance under variable temperature conditions in chickens, Lien et al. [44] compared QTL detected in an F2 chicken population by GWAS using univariate linear mixed model (GEMMA software) and using interval mapping method (QTLMap software). This study showed that eight QTL were detected by QTLMap, 47 were detected by GEMMA and from those, only two were common to both approaches [44].

The detected QTL for GIZZWT on GGA21 (1 Mb) overlapped with the known QTL #96660 (Table 3) that had been localized to a wider region of 3.4 Mb. The use of a high density of SNP and GWAS approach provided us with better resolution in the detection of this QTL, reducing approximately 70% in the size of the associated region.

Four QTL mapped for GIZZWT, GIZZZP and INTES overlapped with QTL previously mapped for fatness, using the method and the exact same birds from Embrapa F2 Chicken Resource population (Table 4). These QTL detected herein may exhibit pleotropic effects with abdominal fat and carcass fat content traits, thus, selection based on genotype for these internal organ traits might affect fat deposition. Moreover, changes in the gizzard weights can influence nutrient availability in the small intestine; intestine lengths are associated with feed efficiency and growth in chickens [18, 19].

We confirmed and refined several known QTL, and we discovered 17 novel QTL associated with LIVP, GIZZWT, GIZZP, LUNG and INTES; from the 20 QTL detected, only three QTL overlapped with known QTL mapped for internal organ traits in chickens (Table 3). These QTL can help to understand the genetic architecture of metabolic important organs that potentially affects feed efficiency and the occurrence of metabolic disorders, such as pulmonary hypertension [5].

### Positional candidate genes

From the 14 PCGs selected, six had been previously reported as candidate genes for internal organ weight regulation in chickens (*GTF2F2*, *SPERT*, *KCTD4*, *HTR2A*, *LCORL* and *LDB2*) [31].

The Ligand dependent nuclear receptor corepressor like (*LCORL*) gene is located within the QTL on GGA4 (76 Mb) associated with GIZZWT, and this region overlapped with a QTL previously associated with the same trait in an F2 Chicken population [31]. This gene has been reported as a candidate gene for body size in horses [45] and for performance and carcass traits in cattle [46], suggesting pleotropic effects. Additionally, *LCORL* gene is located within a haplotype block that harbors the SNP with the highest model frequency, indicating that genetic variants in this gene are in linkage disequilibrium with the QTL. These evidences point to *LCORL* as a candidate gene for GIZZWT in chickens, however, further validation is needed to confirm our findings.

The Protein tyrosine phosphatase, non-receptor type 11 gene (*PTPN11*) is located within the QTL on GGA15 (6 Mb), explaining 0.7% of the genetic variance for LIVP and exhibited GO term annotated for organ growth (GO:0035265). This gene encodes a tyrosine phosphatase (SHP2) that plays a role in biological processes such as cell growth and differentiation [47]. SHP2 affects many biological functions in the gastrointestinal tract [47], and in a study with mice, animals knocked-out for SHP2 exhibited an attenuated hepatocyte proliferation [48].

Within the QTL on GGA1 (rs15488636 - rs13551715) explaining 5.23% of the genetic variance for GIZZWT, two PCGs were identified: *RB1* and *TNFSF11*. The *Retinoblastoma 1* (*RB1*) gene encodes proteins from retinoblastoma (*RB*) family [49] and exhibited GO term annotated for digestive tract development (GO:0048565). In mammals, this family plays a role in the regulation of cell cycle [49] and in the proliferation of pre-adipocytes in chickens [50]. A previous study performed with mouse reported that *RB1* expression can affect the mass of internal organs such as liver, spleen, lungs and heart during postnatal development [51]. The *TNF superfamily member 11* (*TNFSF11*) gene encodes members of the tumor necrosis factor (TNF) family, and this family plays a role in different biological processes such as cell growth and differentiation [52], bone remodeling process and osteoclast differentiation [23]. In a study performed in a broiler chicken population, associations of the *TNFSF11* gene with weight gain, feed conversion and heart weight were identified [23]. Additionally, this gene exhibited GO term for animal organ morphogenesis (GO:0009887). Further studies with this gene may help elucidate its role in gizzard weight regulation.

The *integrin subunit beta 4* (*ITGB4*) gene is located within the QTL on GGA18 (rs315188853 - rs312861933) explaining 2.98% of the genetic variance for GIZZP and encodes ß4 integrin polypeptides [53]. In humans, several studies reported both lethal and nonlethal variants in *ITGB4* gene responsible for epidermal and gastrointestinal disorders [54–58]. Genetic variants in the *ITGB4* gene were associated with the occurrence of pyloric atresia [54, 55], which can lead to a distended stomach in humans [56]. Additionally, this gene exhibited GO term annotated for digestive tract development (GO:0048565).

The *Solute carrier family 6 member 4* (*SLC6A4*) gene is located within the QTL on GGA19 (6 Mb) explaining 1.01% of the genetic variance for INTES and exhibited GO term annotated for negative regulation of organ growth (GO:0046621). This gene belongs to the solute carrier (SLC) family of proteins that encodes membrane-bound transporters [59]. In an expression profile study of the SLC gene family in intestine from the late embryonic to early post-hatch stages, the *SLC6A4* gene exhibited a transient peak of expression during embryonic day of hatch [60]. In humans, *SLC6A4,* also known as *SERT* or *5-HT* gene, is expressed across many regions of the intestine [61]. This gene is expressed in human intestinal epithelial cells and changes in its expression pattern can lead to intestinal disorders [62]. In chickens, intestinal disorders can affect feed efficiency, and consequently, animal performance [18, 19], and this is consistent with the observation that feed intake exhibited a positive genetic correlation with intestine weight (0.92) in a meat-type chicken population [8]. Thus, the *SLC6A4* gene should be thoroughly investigated as a candidate for regulation of intestine length in chickens.

Additionally, three PCGs (*PCDH7*, *NR4A2* and *GPD2*) overlapped with selection signature regions identified in the founder lines of Embrapa F2 Chicken Resource Population [33], suggesting that they are under positive selection, affecting internal organ weights in at least one of the lines (broiler or layer line).

The *Protocadherin 7* (*PCDH7*) gene is located within the QTL on GGA4 (72 Mb) associated with INTES and GIZZWT. This gene is a member of protocadherin gene family and encodes an integral membrane protein that plays a role in cell-cell recognition and adhesion [63]. In a study with humans, *PCDH7* was reported as a hepatic stellate cell surface marker [63] and hepatic stellate cells play important role in the occurrence of liver fibrosis [64]. Thus, the *PCDH7* gene can be responsible for liver disorders. Chen et al. [65] found *PCDH7* with a high expression in human gastric mucosa tissues. This gene is located within a haplotype block that harbor the SNP with the highest model frequency indicating that genetic variants in this gene are in linkage disequilibrium with the QTL. This is the first report showing some evidence to support *PCDH7* as a candidate for regulation of INTES and GIZZWT in chickens. Further functional validation studies are necessary to confirm our findings.

The *Nuclear receptor subfamily 4 group A member 2* (*NR4A2*) and *Glycerol-3-phosphate dehydrogenase 2* (*GPD2*) genes are located within the QTL on GGA7 (36 Mb) associated with INTES. Both genes play a role in glucose homeostasis [66, 67], and consequently, they are involved in lipid metabolism. In mice, changes in glucose levels can lead to a high susceptibility to intestinal barrier dysfunction and enteric infection [68]. These disorders can affect intestinal permeability [69], affecting animal health and performance [70]. Thus, both genes are candidates for INTES regulation in chickens.

Based on the GO terms and the literature information, the 14 genes discussed here are potential candidates for regulation of the weights of internal organs or their proportionate growth, which influences their percentages. Potentially functional genetic variants in these genes may affect expression or can also affect protein function, may leading to changes in the phenotype. Thus, the identification of genetic variants can help to elucidate and support the role of the candidate genes in the regulation of internal organs traits in chickens. Consequently, this knowledge can help to mitigate the occurrence of metabolic disorders that leads to poultry production losses.

### Discovery of potential causative SNP in PCGs

Approximately 81% of the 13,748 SNPs in PCGs were annotated in potentially neutral regions (introns), while 19% were annotated in potentially functional regions (Table 6). Even considered as a potentially neutral region, SNPs in introns can be associated with the phenotype, playing a role in gene expression and alternative splicing regulation as well, affecting the mRNA transport, providing important information to understand the genetic architecture of the trait [71–73]. However, SNPs located in introns were not deeply investigated in this study.

Several studies have reported genetic variants in potentially functional regions (3’and 5′- UTR, down and up-stream from the gene and in exonic regions classified as synonymous and non-synonymous) associated with important traits in chickens [22, 40, 74–77]. However, only a few studies have focused on the investigation of deleterious and high impact variants associated with important traits in chickens [34, 78–80] that can cause loss of protein function or produce a truncated protein. Thus, in the search of potentially functional mutations we focus on predicted deleterious and high impact variants annotated in our PCGs.

Seven predicted deleterious SNPs were identified in three candidate genes (Table 7), all annotated as missense variants; variants that results in amino acid changes but the length is preserved [81]. One SNP (rs312442403) was annotated in the *TNFSF11* gene, a positional candidate for GIZZWT and this genetic variant can be responsible for changes in the tumor necrosis factor (TNF) family affecting cell growth and differentiation.

Three SNPs (g.36224286 > C/T, g.36225242 > G/T and g.36225278 > C/T) were annotated in the *NR4A2* gene, a positional candidate gene for INTES and these genetic variants might be responsible for changes in glucose levels driving the occurrence of intestinal disorders. Three other SNPs (rs313279811, rs316340790 and rs732847450) were annotated in the *ITGB4* gene, positional candidate for GIZZP, and these genetic variants can result in epidermal and gastrointestinal disorders. It is important to highlight that these mutations are potentially functional and further association and functional studies should be performed to validate their role in phenotype regulation. No high impact variants were detected in our PCGs.

From all the PCGs, four (*RB1, HTR2A, NR4A2* and *GPD2*) were already selected as candidates for regulating fat deposition in the same population in a study by Moreira et al. [32], suggesting that they may exhibit pleiotropic effects with fat deposition traits.

## Conclusions

High genomic heritability estimates were reported for some traits indicating that they can respond successfully to selection based on genotypes. QTL were detected for LIVP, GIZZWT, GIZZP, LUNGP and INTES, across the whole-genome. Within these QTL, PCGs involved in biological processes that might regulate internal organ growth and morphogenesis, digestive tract development, and metabolic disorders and diseases were identified. These biological processes may influence feed efficiency and also the occurrence of metabolic disorders leading to losses in poultry production. Potentially functional genetic variants annotated in PCGs were identified and these mutations can responsible for phenotypic changes. Further validation studies can elucidate their role in internal organ traits regulation, allowing its possible application in poultry breeding programs aiming to select chickens with higher feed efficiency and lower susceptibility for metabolic disorders, thus improving chicken production.

## Methods

All experimental protocols related to animal experimentation in this study were performed in agreement with resolution number 011/2010 approved by the Embrapa Swine and Poultry Ethics Committee on Animal Utilization (CEUA) in Concordia, Santa Catarina State – South of Brazil. These protocols are in agreement with the rules of the National Council of Animal Experimentation Control (CONCEA) to ensure compliance with international guidelines for animal welfare. The population, genotypes and methods described are the same as those adopted by Moreira et al. [32] in a previous GWAS performed by our group for fat deposition traits.

### Chicken population

Embrapa F_2_ Chicken Resource Population used in this study was developed by the Embrapa Swine and Poultry National Research Center, for QTL mapping and genomic studies [6, 38], from reciprocal crosses between one layer line (known as CC) and one broiler line (known as TT). The grandparental lines had previously undergone multi-trait selection over many generations. The CC line had been selected for improved egg production, egg weight, feed conversion, viability, sexual maturity, fertility, hatchability, egg quality and for low body weight, while the TT line had been selected for improved body weight, feed conversion, carcass and breast yield, viability, fertility, hatchability, and for reduced abdominal fat and metabolic syndromes. More details about the Embrapa F_2_ Chicken Resource Population are in Nones et al. [6] and Rosário et al. [38]. A total of 28 parental (from the broiler and layer lines), five F_1_ and 496 F_2_-TCTC chickens were genotyped with a 600 K Axiom Chicken Genotyping Array, which contains SNPs segregating for different chicken lines [27].

The chickens and genotypes are the same as those adopted by Moreira et al. [32] in a previous GWAS performed in the same population, that was capable to detect reliable QTLs for fat deposition traits. Moreover, this sample size is in agreement with other previous GWAS that utilized 600 K Axiom Chicken Genotyping Array and also detected reliable QTLs for production traits [82, 83].

### Phenotypic measurement

After 6 h of fasting, chickens at 42 days of age had their live body weight measured (BW42) and were then euthanized by cervical dislocation. A blood sample from each chicken was immediately collected for subsequent DNA extraction, then the carcasses were eviscerated, the internal organs (heart, lungs, liver and gizzard) were weighted, the intestine length was measured [5] and the carcasses were cooled. The percentage traits for each of the organs were calculated by dividing their weight by BW42 and multiplying by 100. More details about measurement of internal organs in the F_2_ population are in Moura et al. [5].

### DNA extraction, genotyping and quality control

The genomic DNA from 529 chickens was extracted from blood samples with DNAzol® protocol. The DNA integrity was evaluated in agarose gel (1%), quantified in a NanoDrop® spectrophotometer (Thermo Fisher Scientific) then diluted to the final concentration of 20 ng/μL. All subsequent DNA preparation steps for genotyping were performed following recommended Affymetrix protocols. The samples were genotyped with the 600 K Axiom Chicken Genotyping Array [27].

Samples and genotypes quality control were performed using Affymetrix Power Tools v1.17.0 (APT) software. Samples with DishQC ≤0.82 and call rate ≤ 90% as well as SNPs with call rate ≤ 98%, minor allele frequency (MAF) ≤ 2% and those not annotated in autosomal chromosomes, were removed from the dataset. The genome position of SNPs was taken to be that on the *Gallus_gallus-5.0* chicken assembly (NCBI). At any particular locus, the small number of missing genotypes were replaced by their average covariate value representing the number of copies of one of the alleles at that locus across all the individuals with an observed genotype at that locus [84].

### Descriptive statistics and heritability

Descriptive statistics (average and standard deviation) for each trait were calculated using basic functions in R software (http://www.r-project.org/). The variance components reported (genetic, residual and total variance) were obtained from the means of the posterior distributions of those components, obtained by fitting a Bayes B model in GenSel software [85]. The posterior distribution for heritability (the ratio of genetic variance divided by total variance) was constructed from each sample of the posterior distributions of the variance components, and the posterior mean is reported as the estimate of the genomic heritability.

### Genome-wide association analysis

Informative and reliable SNPs (after quality control filters) were kept for GWAS analysis with a Bayesian approach in GenSel software [85]. Initially, Bayes C was used to estimate the variance components and then, genetic and residual variances were used as *priors* to run a Bayes B model for inference about genomic effects [78, 84]. The mathematical model was:

$$ \boldsymbol{y}=\boldsymbol{Xb}+\sum \limits_{j=1}^k{\boldsymbol{a}}_j{\beta}_j{\delta}_j+\boldsymbol{e}, $$

where, ***y*** represents the vector of phenotypic values for one trait, ***X*** is the incidence matrix for fixed effects, ***b*** is the vector of fixed effects, *K* is the number of SNPs, **a**_***j***_ is the column vector representing the SNP as a covariate for locus _j_ coded with the number of B alleles, *βj* is the random substitution effect for locus *j,* assumed to be normally distributed *N* (0, *σ*^2^_*βi*_) when *δj* = 1 but *βj* = 0 when *δj* = 0, with *δj* being a random variable 0/1, indicating the absence (with probability π) or presence (with probability 1-π) of locus *j* in the model equation and ***e*** is the residual associated with the analysis. In the model, sex and hatch were included as fixed effects and BW42 as a fixed covariate for weight traits. We tested the significance of the effects using a linear regression model in R software (http://www.r-project.org/).

Parameterization of the BayesB model assumed π = 0.9988 and involved obtaining 41,000 Markov Chain Monte Carlo (MCMC) samples from which the first 1000 samples were discarded. The genome was partitioned into 943 non-overlapping 1 Mb windows, based on genomic locations of SNP provided in a map file. In order to select associated windows, we assumed that each window was expected to explain approximately 0.1060% of the genetic variance (100%/943) based on an infinitesimal model [30, 83]. Windows explaining five-fold more than expected (0.53%) were considered biologically significant and only those windows were used as the basis for identification of PCGs.

### Overlap with known QTL

We checked the overlap of associated genomic windows with known QTL for internal organs available in the Chicken QTL database – release 35 [29], accessed in July, 2018. The overlaps were checked using QTL coordinates according to the Gallus_gallus-5.0 (NCBI) chicken genome assembly, available in the BED file. We excluded those QTL without genomic positions and also those not properly annotated (provisionally annotated between 0 and 100 bp), leaving 16 well annotated QTL for overlap analysis. Genomic windows that did not overlap with known QTL were considered novel.

We checked the overlap between our associated genomic windows and the QTL for internal organ weights recently reported from the analysis of a Chinese F2 population [31]. Additionally, we investigated the overlap between the QTL detected herein and QTL detected for fatness traits, using the same method and population [32].

### Identification of positional candidate genes

We considered the flanking positions from the genomic windows associated with internal organ traits in order to search for PCGs. An entire gene list was obtained using Ensembl BioMart tool [86, 87]. In order to refine that gene list and select PCGs, we adopted two criteria: (1) genes located within haplotype blocks harboring the SNP with the highest model frequency in each associated genomic window (the haplotype blocks detection was performed using PLINK v.1.9 [88] software with default parameters); and (2) genes that overlapped with selection signature regions identified in a previous study [33], which evaluated 28 grandparental chickens from the two lines that generated the F_2_ population analyzed in this study.

The previous study [33] used whole genome sequences to identify genetic variants and applied Fst method [33, 89] to estimate the divergence between populations to identify regions under selection (TT vs. CC lines). We used the CrossMap tool (http://crossmap.sourceforge.net/) to convert selection signature region coordinates from *Gallus_gallus*-4.0 to the *Gallus_gallus*-5.0 chicken genome assembly (NCBI).

Additionally, we investigated genes that exhibit biological processes from GO terms directly related to internal organs growth and development. Further, the genes selected in each step were investigated in different databases (NCBI, OMIM), and in the literature, to support/refute their selection as PCGs.

### Detection of potential functional mutations

We searched for deleterious and high impact SNPs annotated in PCGs to identify potentially functional mutations. A dataset of approximately 13 million reliable SNPs were initially identified in the whole genome as a result of sequencing 28 grandparental chickens from the Embrapa F_2_ Chicken Resource Population, and after filtration, approximately 14 million SNPs were further utilized for the search of SNPs annotated in PCGs. A detailed description of the sequencing, SNP calling, and filtering criteria are available in Boschiero et al. [43].

The filtered SNPs dataset was annotated using Variant Effect Predictor (VEP) tool [90]. For the variants located in coding regions, the VEP tool was also used to calculate the SIFT (sorting intolerant from tolerant) score. This score is an assessment of the level of conservation in homologous protein sequences, used to predict whether amino acid changes caused by SNPs are tolerant nor not (may affect the function of the gene product). Coding SNPs with SIFT scores < 0.05 are predicted as deleterious, whereas coding SNPs with SIFT scores ≥0.05 are predicted as tolerated [91].

Prediction of the putative variant impact was also performed using the VEP tool [90]. All variants annotated as transcript ablation, splice acceptor, splice donor, stop gained, frameshift, stop loss, start lost and transcript amplification which may cause protein truncation, loss of function or trigger nonsense mediated decay were predicted as high impact variants (https://www.ensembl.org/info/genome/variation/prediction/predicted_data.html) [78].

## Additional files

Additional file 1: An excel file with the characterization of all the 943 1-Mb SNP windows analyzed, including the percentage of the genetic variance explained by each one. (XLS 893 kb)

Additional file 2: List with the Ensembl Gene ID and the respective gene names of the 756 genes annotated within the QTL detected. (TXT 17 kb)

Additional file 3: Genomic windows associated with internal organ traits overlapping with selection signature regions detected in the founder chickens of the Embrapa F_2_ Chicken Resource Population. (DOCX 90 kb)

Additional file 4: Characterization of the genomic windows and their respective haplotype blocks. (DOCX 20 kb)

## Abbreviations

DAVID: Database for Annotation, Visualization and Integrated Discovery software
GIZZP: Gizzard Weight as a Percentage of Body Weight
GIZZWT: Gizzard Weight
GWAS: Genome-Wide Association Study
HEARTP: Heart Weight as a Percentage of Body Weight
HWT: Heart Weight
INTES: Intestine Length
LIVP: Liver Weight as a Percentage of Body Weight
LIVWT: Liver Weight
LUNGP: Lung Weight as a Percentage of Body Weight
LUNGWT: Lung Weight
MAF: Minor Allele Frequency
MCMC: Markov chain Monte Carlo
NCBI: National Center for Biotechnology Information
OMIM: Online Mendelian Inheritance in Man
PCG: Positional Candidate Gene
PPA: Posterior Probability of Association
QTL: Quantitative Trait Locus
SNP: Single Nucleotide Polymorphism

## References

[1] Berri C, Wacrenier N, Millet N, Le Bihan-Duval E (2001). Effect of selection for improved body composition on muscle and meat characteristics of broilers from experimental and commercial lines. Poult Sci.

[2] Baéza E, Le Bihan-Duval E (2013). Chicken lines divergent for low or high abdominal fat deposition: a relevant model to study the regulation of energy metabolism. Animal.

[3] Jennen DGJ (2004). Vereijken a LJ, Bovenhuis H, Crooijmans RPM a, Veenendaal a, van der Poel JJ, et al. detection and localization of quantitative trait loci affecting fatness in broilers. Poult Sci.

[4] Wang SZ, Hu XX, Wang ZP, Li XC, Wang QG, Wang YX (2012). Quantitative trait loci associated with body weight and abdominal fat traits on chicken chromosomes 3, 5 and 7. Genet Mol Res.

[5] Moura ASAMTAMT, Ledur MC, Boschiero C, Nones K, Pinto LFBB, Jaenisch FRFF (2016). Quantitative trait loci with sex-specific effects for internal organs weights and hematocrit value in a broiler-layer cross. J Appl Genet.

[6] Nones K, Ledur MC, Ruy DC, Baron EE, Melo CMR, Moura ASAMT (2006). Mapping QTLs on chicken chromosome 1 for performance and carcass traits in a broiler x layer cross. Anim Genet.

[7] Burt DW (2002). Applications of biotechnology in the poultry industry. Worlds Poult Sci J.

[8] Gaya LG, Ferraz JBS, Rezende FM, Mourao GB, Mattos EC, Eler JP (2006). Heritability and Genetic Correlation Estimates for Performance and Carcass and Body Composition Traits in a Male Broiler Line. Poult Sci.

[9] Schmidt CJ, Persia ME, Feierstein E, Kingham B (2009). Saylor WW. Comparison of a modern broiler line and a heritage line unselected since the 1950s. Poult Sci.

[10] Julian RJ (1998). Rapid growth problems: ascites and skeletal deformities in broilers. Poult Sci.

[11] Fernandes Do Rosário M, Neves Da Silva MA, Augusto A, Coelho D, José V, Savino M. Síndrome ascítica em frangos de corte: uma revisão sobre a fisiologia, avaliação e perspectivas Ascitic syndrome in broiler chickens: a review about physiology, evaluation and perspectives. Ciência Rural. 2004; cited 2018 Aug 4; Available from: http://www.scielo.br/pdf/cr/v34n6/a51v34n6.pdf.

[12] Olkowski AA, Duke T, Wojnarowicz C (2005). The aetiology of hypoxaemia in chickens selected for rapid growth. Comp Biochem Physiol Part A Mol Integr Physiol.

[13] Tankson JD, Thaxton JP, Vizzier-Thaxton Y (2001). Pulmonary hypertension syndrome in broilers caused by *Enterococcus faecalis*. Infect Immun.

[14] Olkowski AA, Classen HL (1998). High incidence of cardiac arrhythmias in broiler chickens. Zentralbl Veterinarmed A.

[15] Olkowski AA, Classen HL, Riddell C, Bennett CD (1997). A Study of Electrocardiographic Patterns in a Population of Commercial Broiler Chickens. Vet Res Commun.

[16] Olkowski AA (2007). Pathophysiology of Heart Failure in Broiler Chickens: Structural, Biochemical, and Molecular Characteristics. Poult Sci.

[17] Olkowski AA, Wojnarowicz C, Nain S, Ling B, Alcorn JM, Laarveld B (2008). A study on pathogenesis of sudden death syndrome in broiler chickens. Res Vet Sci.

[18] de Verdal H, Narcy A, Bastianelli D, Chapuis H, Même N, Urvoix S (2011). Improving the efficiency of feed utilization in poultry by selection. 1. Genetic parameters of anatomy of the gastro-intestinal tract and digestive efficiency. BMC Genet.

[19] Li S, Wang X, Qu L, Dou T, Ma M, Shen M (2018). Genome-wide association studies for small intestine length in an F _2_ population of chickens. Ital J Anim Sci.

[20] Scheele CW (1997). Pathological changes in metabolism of poultry related to increasing production levels. Vet Q.

[21] Mignon-Grasteau S, Rideau N, Gabriel I, Chantry-Darmon C, Boscher M-Y, Sellier N (2015). Detection of QTL controlling feed efficiency and excretion in chickens fed a wheat-based diet. Genet Sel Evol.

[22] Boschiero C, Jorge EC, Ninov K, Nones K, do Rosário MF, Coutinho LL (2013). Association of IGF1 and KDM5A polymorphisms with performance, fatness and carcass traits in chickens. J Appl Genet.

[23] Grupioni NV, Stafuzza NB, Carvajal AB, Ibelli AMG, Peixoto JO, Ledur MC, et al. Association of RUNX2 and TNFSF11 genes with production traits in a paternal broiler line. Genet Mol Res. 2017;16:gmr16019443. 10.4238/gmr16019443 28340265

[24] Gao Y, Du ZQ, Wei WH, Yu XJ, Deng XM, Feng CG (2009). Mapping quantitative trait loci regulating chicken body composition traits. Anim Genet.

[25] Navarro P, Visscher PM, Knott SA, Burt DW, Hocking PM, Haley CS (2005). Mapping of quantitative trait loci affecting organ weights and blood variables in a broiler layer cross. Br Poult Sci.

[26] Ek W, Strömstedt L, Wahlberg P, Siegel P, Andersson L, Carlborg Ö (2010). Genetic analysis of metabolic traits in an intercross between body weight-selected chicken lines. Physiol Genomics.

[27] Kranis A, Gheyas AA, Boschiero C, Turner F, Yu L, Smith S (2013). Development of a high density 600K SNP genotyping array for chicken. BMC Genomics.

[28] Zhou X, Stephens M (2012). Genome-wide efficient mixed-model analysis for association studies. Nat Genet.

[29] Hu Z-L, Park CA, Wu X-L, Reecy JM (2013). Animal QTLdb: an improved database tool for livestock animal QTL/association data dissemination in the post-genome era. Nucleic Acids Res.

[30] Onteru SK, Gorbach DM, Young JM, Garrick DJ, Dekkers JCM, Rothschild MF. Whole Genome Association Studies of Residual Feed Intake and Related Traits in the Pig. Liu Z, editor. PLoS One. 2013;8:e61756. [cited 2017 Nov 7] Available from: http://dx.plos.org/10.1371/journal.pone.0061756 10.1371/journal.pone.0061756 PMC3694077 23840294

[31] Dou T, Shen M, Ma M, Qu L, Li Y, Hu Y, et al. Genetic architecture and candidate genes detected for chicken internal organ weight with a 600 K SNP array. Asian-Australasian J Anim Sci. 2018;32(3):341–9. 10.5713/ajas.18.0274 PMC6409475 30056651

[32] Moreira GCM, Boschiero C, Cesar ASM, Reecy JM, Godoy TF, Pértille F (2018). Integration of genome wide association studies and whole genome sequencing provides novel insights into fat deposition in chicken. Sci Rep.

[33] Boschiero C, Moreira GCM, Gheyas AA, Godoy TF, Gasparin G, Mariani PDSC (2018). Genome-wide characterization of genetic variants and putative regions under selection in meat and egg-type chicken lines. BMC Genomics.

[34] Moreira GCM, Godoy TF, Boschiero C, Gheyas A, Gasparin G, Andrade SCS (2015). Variant discovery in a QTL region on chromosome 3 associated with fatness in chickens. Anim Genet.

[35] Kumar Prateek, Henikoff Steven, Ng Pauline C (2009). Predicting the effects of coding non-synonymous variants on protein function using the SIFT algorithm. Nature Protocols.

[36] Venturini G.C., Cruz V.A.R., Rosa J.O., Baldi F., El Faro L., Ledur M.C., Peixoto J.O., Munari D.P. (2014). Genetic and phenotypic parameters of carcass and organ traits of broiler chickens. Genetics and Molecular Research.

[37] Marchesi JAP, Buzanskas ME, Cantão ME, Ibelli AMG, Peixoto JO, Joaquim LB, et al. Relationship of runs of homozygosity with adaptive and production traits in a paternal broiler line. Animal. 2017:1–9 [cited 2018 Mar 24] Available from: http://www.ncbi.nlm.nih.gov/pubmed/29065939. 10.1017/S1751731117002671 29065939

[38] MF do R, Ledur MC, ASAMT M, Coutinho LL, AAF G (2009). Genotypic characterization of microsatellite markers in broiler and layer selected chicken lines and their reciprocal F1s. Sci Agric.

[39] Pértille F, Zanella R, Felício AM, Ledur MC, Peixoto JO, Coutinho LL (2015). Identification of polymorphisms associated with production traits on chicken (*Gallus gallus*) chromosome 4. Genet Mol Res.

[40] Pértille F, Moreira GCM, Zanella R, Nunes JR, Boschiero C, Rovadoscki GA (2017). Genome-wide association study for performance traits in chickens using genotype by sequencing approach. Sci Rep.

[41] Tran T-S, Narcy A, Carré B, Gabriel I, Rideau N, Gilbert H (2014). Detection of QTL controlling digestive efficiency and anatomy of the digestive tract in chicken fed a wheat-based diet. Genet Sel Evol.

[42] Faveri JC, Pinto LFB, Pedrosa VB, Ledur MC, Faveri JC, Pinto LFB (2016). Parâmetros genéticos e efeitos de sexo e cruzamento recíproco sobre características de interesse econômico em aves F2. Arq Bras Med.

[43] Park Hee-Bok, Heo Kang-Nyeong, Kang Bo-Seok, Jo Cheorun, Lee Jun Heon (2013). Power of Variance Component Linkage Analysis to Identify Quantitative Trait Locus in Chickens. Journal of Animal Science and Technology.

[44] Lien C-Y, Tixier-Boichard M, Wu S-W, Wang W-F, Ng CS, Chen C-F (2017). Detection of QTL for traits related to adaptation to sub-optimal climatic conditions in chickens. Genet Sel Evol.

[45] Metzger J, Schrimpf R, Philipp U, Distl O (2013). Expression Levels of LCORL Are Associated with Body Size in Horses. PLoS One.

[46] Lindholm-Perry AK, Sexten AK, Kuehn LA, Smith TP, King DA, Shackelford SD (2011). Association, effects and validation of polymorphisms within the NCAPG - LCORL locus located on BTA6 with feed intake, gain, meat and carcass traits in beef cattle. BMC Genet.

[47] Coulombe G, Rivard N (2016). New and Unexpected Biological Functions for the Src-Homology 2 Domain-Containing Phosphatase SHP-2 in the Gastrointestinal Tract. Cell Mol Gastroenterol Hepatol.

[48] Bard-Chapeau EA, Yuan J, Droin N, Long S, Zhang EE, Nguyen TV (2006). Concerted Functions of Gab1 and Shp2 in Liver Regeneration and Hepatoprotection. Mol Cell Biol.

[49] Henley SA, Dick FA (2012). The retinoblastoma family of proteins and their regulatory functions in the mammalian cell division cycle. Cell Div.

[50] Wang Y-X, Wang H-X, Na W, Qin F-Y, Zhang Z-W, Dong J-Q, et al. Retinoblastoma 1 (RB1) modulates the proliferation of chicken preadipocytes. bioRxiv. 2018;341453 [cited 2018 Sep 27] Available from: https://www.biorxiv.org/content/early/2018/06/07/341453. 10.1080/00071668.2019.1584792 30784300

[51] Nikitin AY, Shan B, Flesken-Nikitin A, Chang KH, Lee WH (2001). The retinoblastoma gene regulates somatic growth during mouse development. Cancer Res.

[52] Schneider Pascal, MacKay Fabienne, Steiner Véronique, Hofmann Kay, Bodmer Jean-Luc, Holler Nils, Ambrose Christine, Lawton Pornsri, Bixler Sarah, Acha-Orbea Hans, Valmori Danila, Romero Pedro, Werner-Favre Christiane, Zubler Rudolph H., Browning Jeffrey L., Tschopp Jürg (1999). BAFF, a Novel Ligand of the Tumor Necrosis Factor Family, Stimulates B Cell Growth. The Journal of Experimental Medicine.

[53] Takizawa Y, Shimizu H, Nishikawa T, Hatta N, Pulkkinen L, Uitto J (1997). Novel ITGB4 mutations in a patient with junctional epidermolysis bullosa-pyloric atresia syndrome and altered basement membrane zone immunofluorescence for the alpha6beta4 integrin. J Invest Dermatol.

[54] Pulkkinen L, Kim DU, Uitto J (1998). Epidermolysis bullosa with pyloric atresia: novel mutations in the beta4 integrin gene (ITGB4). Am J Pathol.

[55] Pulkkinen L, Rouan F, Bruckner-Tuderman L, Wallerstein R, Garzon M, Brown T (1998). Novel ITGB4 Mutations in Lethal and Nonlethal Variants of Epidermolysis Bullosa with Pyloric Atresia: Missense versus Nonsense. Am J Hum Genet.

[56] Azarian M, Dreux S, Vuillard E, Meneguzzi G, Haber S, Guimiot F (2006). Prenatal diagnosis of inherited epidermolysis bullosa in a patient with no family history: a case report and literature review. Prenat Diagn.

[57] Pyloric BS (2013). Atresia Type II. J neonatal Surg.

[58] Chung HJ, Uitto J (2010). Epidermolysis bullosa with pyloric atresia. Dermatol Clin.

[59] He L, Vasiliou K, Nebert DW (2009). Analysis and update of the human solute carrier (SLC) gene superfamily. Hum Genomics.

[60] Li H, Gilbert ER, Zhang Y, Crasta O, Emmerson D, Webb KE (2008). Expression profiling of the solute carrier gene family in chicken intestine from the late embryonic to early post-hatch stages. Anim Genet.

[61] Gill RK, Pant N, Saksena S, Singla A, Nazir TM, Vohwinkel L (2008). Function, expression, and characterization of the serotonin transporter in the native human intestine. Am J Physiol Liver Physiol.

[62] Gill RK, Anbazhagan AN, Esmaili A, Kumar A, Nazir S, Malakooti J (2011). Epidermal growth factor upregulates serotonin transporter in human intestinal epithelial cells via transcriptional mechanisms. Am J Physiol Liver Physiol.

[63] Zhang DY, Goossens N, Guo J, Tsai M-C, Chou H-I, Altunkaynak C (2016). A hepatic stellate cell gene expression signature associated with outcomes in hepatitis C cirrhosis and hepatocellular carcinoma after curative resection. Gut.

[64] Zhang C-Y, Yuan W-G, He P, Lei J-H, Wang C-X (2016). Liver fibrosis and hepatic stellate cells: Etiology, pathological hallmarks and therapeutic targets. World J Gastroenterol.

[65] Chen H-F, Ma R-R, He J-Y, Zhang H, Liu X-L, Guo X-Y (2017). Protocadherin 7 inhibits cell migration and invasion through E-cadherin in gastric cancer. Tumor Biol.

[66] Han Y-F, Cao G-W (2012). Role of nuclear receptor NR4A2 in gastrointestinal inflammation and cancers. World J Gastroenterol.

[67] Madiraju AK, Erion DM, Rahimi Y, Zhang X-M, Braddock DT, Albright RA (2014). Metformin suppresses gluconeogenesis by inhibiting mitochondrial glycerophosphate dehydrogenase. Nature.

[68] Thaiss CA, Levy M, Grosheva I, Zheng D, Soffer E, Blacher E (2018). Hyperglycemia drives intestinal barrier dysfunction and risk for enteric infection. Science.

[69] De Santis S, Cavalcanti E, Mastronardi M, Jirillo E, Chieppa M (2015). Nutritional Keys for Intestinal Barrier Modulation. Front Immunol.

[70] Celi P, Verlhac V, Pérez Calvo E, Schmeisser J, Kluenter A-M. Biomarkers of gastrointestinal functionality in animal nutrition and health. Anim Feed Sci Technol. 2018; [cited 2018 Sep 28]; Available from: https://www.sciencedirect.com/science/article/pii/S0377840118302438.

[71] Pinsonneault JK, Frater JT, Kompa B, Mascarenhas R, Wang D, Sadee W (2017). Intronic SNP in ESR1 encoding human estrogen receptor alpha is associated with brain ESR1 mRNA isoform expression and behavioral traits. PLoS One.

[72] Jo B-S, Choi SS (2015). Introns: The Functional Benefits of Introns in Genomes. Genomics Inform.

[73] Berulava T, Horsthemke B (2010). The obesity-associated SNPs in intron 1 of the FTO gene affect primary transcript levels. Eur J Hum Genet.

[74] Ou JT, Tang SQ, Sun DX, Zhang Y (2009). Polymorphisms of three neuroendocrine-correlated genes associated with growth and reproductive traits in the chicken. Poult Sci.

[75] Xie L, Luo C, Zhang C, Zhang R, Tang J, Nie Q (2012). Genome-wide association study identified a narrow chromosome 1 region associated with chicken growth traits. Liu Z, editor. PLoS One.

[76] Yan G, Qiao R, Zhang F, Xin W, Xiao S, Huang T (2017). Imputation-Based Whole-Genome Sequence Association Study Rediscovered the Missing QTL for Lumbar Number in Sutai Pigs. Sci Rep.

[77] Felício AM, Boschiero C, Balieiro JCC, Ledur MC, Ferraz JBS, Michelan Filho T (2013). Identification and association of polymorphisms in CAPN1 and CAPN3 candidate genes related to performance and meat quality traits in chickens. Genet Mol Res.

[78] Moreira GCM, Boschiero C, Cesar ASM, Reecy JM, Godoy TF, Trevisoli PA (2018). A genome-wide association study reveals novel genomic regions and positional candidate genes for fat deposition in broiler chickens. BMC Genomics.

[79] Derks MFL, Megens H-J, Bosse M, Visscher J, Peeters K, Bink MCAM (2018). A survey of functional genomic variation in domesticated chickens. Genet Sel Evol.

[80] Wu Z, Derks MFL, Dibbits B, Megens H-J, Groenen MAM, RPMA C. A Novel Loss-of-Function Variant in Transmembrane Protein 263 (TMEM263) of Autosomal Dwarfism in Chicken. Front Genet. 2018; [cited 2018 Aug 2];9:193. Available from: http://www.ncbi.nlm.nih.gov/pubmed/29930570. 10.3389/fgene.2018.00193 PMC6001002 29930570

[81] Calculated consequences [Internet]. [cited 2019 Jun 3]. Available from: https://www.ensembl.org/info/genome/variation/prediction/predicted_data.html. Accessed July 2018.

[82] Van Goor A, Bolek KJ, Ashwell CM, Persia ME, Rothschild MF, Schmidt CJ (2015). Identification of quantitative trait loci for body temperature, body weight, breast yield, and digestibility in an advanced intercross line of chickens under heat stress. Genet Sel Evol.

[83] Van Goor A, Ashwell CM, Persia ME, Rothschild MF, Schmidt CJ, Lamont SJ (2016). Quantitative trait loci identified for blood chemistry components of an advanced intercross line of chickens under heat stress. BMC Genomics.

[84] Cesar AS, Regitano LC, Mourão GB, Tullio RR, Lanna DP, Nassu RT (2014). Genome-wide association study for intramuscular fat deposition and composition in Nellore cattle. BMC Genet.

[85] Garrick DJ, Fernando RL (2013). Implementing a QTL detection study (GWAS) using genomic prediction methodology. Methods Mol Biol.

[86] Flicek Paul, Ahmed Ikhlak, Amode M. Ridwan, Barrell Daniel, Beal Kathryn, Brent Simon, Carvalho-Silva Denise, Clapham Peter, Coates Guy, Fairley Susan, Fitzgerald Stephen, Gil Laurent, García-Girón Carlos, Gordon Leo, Hourlier Thibaut, Hunt Sarah, Juettemann Thomas, Kähäri Andreas K., Keenan Stephen, Komorowska Monika, Kulesha Eugene, Longden Ian, Maurel Thomas, McLaren William M., Muffato Matthieu, Nag Rishi, Overduin Bert, Pignatelli Miguel, Pritchard Bethan, Pritchard Emily, Riat Harpreet Singh, Ritchie Graham R. S., Ruffier Magali, Schuster Michael, Sheppard Daniel, Sobral Daniel, Taylor Kieron, Thormann Anja, Trevanion Stephen, White Simon, Wilder Steven P., Aken Bronwen L., Birney Ewan, Cunningham Fiona, Dunham Ian, Harrow Jennifer, Herrero Javier, Hubbard Tim J. P., Johnson Nathan, Kinsella Rhoda, Parker Anne, Spudich Giulietta, Yates Andy, Zadissa Amonida, Searle Stephen M. J. (2012). Ensembl 2013. Nucleic Acids Research.

[87] Kinsella R. J., Kahari A., Haider S., Zamora J., Proctor G., Spudich G., Almeida-King J., Staines D., Derwent P., Kerhornou A., Kersey P., Flicek P. (2011). Ensembl BioMarts: a hub for data retrieval across taxonomic space. Database.

[88] Purcell S, Neale B, Todd-Brown K, Thomas L, Ferreira MAR, Bender D (2007). PLINK: A Tool Set for Whole-Genome Association and Population-Based Linkage Analyses. Am J Hum Genet.

[89] Weir BS, Cockerham CC (1984). Estimating F-Statistics for the Analysis of Population Structure. Evolution (N Y).

[90] McLaren W, Pritchard B, Rios D, Chen Y, Flicek P, Cunningham F (2010). Deriving the consequences of genomic variants with the Ensembl API and SNP Effect Predictor. Bioinformatics.

[91] Ng PC, SIFT HS (2003). Predicting amino acid changes that affect protein function. Nucleic Acids Res.

