# Supplementary material for: Genome-wide association scan for QTL and their positional candidate genes associated with internal organ traits in chickens
Source: BMC Genomics. 2019 Aug 22;20:669. doi: 10.1186/s12864-019-6040-3 (PMC6704653; doi:10.1186/s12864-019-6040-3)
Supplement: Supplementary file 3 — Genomic windows associated with internal organ traits overlapping with selection signature regions detected in the founder chickens of the Embrapa F2 Chicken Resource Population. (DOCX 90 kb) [file 12864_2019_6040_MOESM3_ESM.docx]

Additional file 3– Genomic windows associated with internal organ traits overlapped with selection signature regions detected in the founder birds of the Embrapa F_2_ Chicken Resource Population.

| **Genomic Windows** | | **Selection signature regions** | |
| --- | --- | --- | --- |
| **GGA^1^** | **Traits** | **Start-end position^1^** | **Annotated genes within the selection signature regions^2^** |
| 1_65 | GIZZP | 65,021,246 – 65,041,245 | *PDE3A* |
| 4_71 | INTES, GIZZWT | 71,264,381 – 71,294,374 | -- |
|  |  | 71,474,403 – 71,494,401 | ENSGALG00000030680 |
|  |  | 71,711,975 – 71,770,421 |  |
| 4_72 | INTES, GIZZWT | 71,982,374 – 72,062,369 | ENSGALG00000031185, *PCDH7* |
|  |  | 72,082,350 – 72,142,387 | *PCDH7* |
|  |  | 72,670,124 – 72,690,125 | -- |
| 7_34 | INTES | 34,676,748 – 34,706,655 | *ACVR2A* |
| 7_36 | INTES | 36,220,480 – 36,270,797 | *NR4A2, GPD2* |
| 15_11 | INTES | 11,370,515 – 11,390,513 | *NOS1* |
| 18_5 | GIZZP | 5,371,357 – 5,421,358 | *CA10* |
| 18_6 | GIZZP | 6,010,064 – 6,030,062 | *HLF*, ENSGALG00000039459, ENSGALG00000039939, *MMD* |
|  |  |  |  |
|  |  |  |  |
|  |  |  |  |
| 19_6 | INTES | 6,460,571 – 6,540,572 | *TTC19*, *NCOR1*, ENSGALG00000004492 |
|  |  |  |  |
|  |  |  |  |

GIZZWT: gizzard weight; GIZZP: gizzard percentage; INTES: intestine length.

^1^ Map position based on Gallus_gallus-5.0 assembly (NCBI).

^2^ Ensembl gene name and ID based on Galgal5 (*Ensembl Genes 93 Database*).
