# Supplementary material for: Genome-wide association scan for QTL and their positional candidate genes associated with internal organ traits in chickens
Source: BMC Genomics. 2019 Aug 22;20:669. doi: 10.1186/s12864-019-6040-3 (PMC6704653; doi:10.1186/s12864-019-6040-3)
Supplement: Supplementary file 4 — Characterization of the genomic windows and their respective haplotype blocks. (DOCX 20 kb) [file 12864_2019_6040_MOESM4_ESM.docx]

Additional file 4 – Characterization of the genomic windows and their respective haplotype blocks.

| **Trait** | **GGA_Mb^1^** | **SNP ID** | **Haplotype Blocks** | | |
| --- | --- | --- | --- | --- | --- |
|  |  |  | **Start-end position^1^** | **Size (kb)** | **Annotated genes within haplotype blocks^2^** |
| LIVP | 2_127 | AX-75998322 | 127,294,322 – 127,299,812 | 5.49 | -- |
|  | 15_6 | AX-75841007 | 6,746,490 – 6,802,917 | 56.43 | *FOXN4, MYO1H, KCTD10, UBE3B* |
| GIZZWT | 1_81 | AX-75530136 | -- | -- | -- |
|  | 1_166 | AX-75327108 | 166,387,653 – 166,390,342 | 2.69 | -- |
|  | 1_167 | AX-75329630 | -- | -- | -- |
|  | 1_168 | AX-75331111 | -- | -- | -- |
|  | 1_169 | AX-75333752 | 169,118,122 – 169,140,070 | 21.95 | *CDADC1* |
|  | 4_71 | AX-76721942 | 71,901,345 – 72,101,291 | 199.95 | *PCDH7*, ENSGALG00000031185 |
|  | 4_72 | AX-76722852 | 72,417,314 – 72,510,041 | 92.73 | ENSGALG00000042737 |
|  | 4_76 | AX-76730299 | 76,264,926 – 76,399,799 | 134.87 | *LCORL* |
|  | 18_7 | AX-75908542 | 7,469,286 – 7,485,829 | 16.54 | *PRKCA* |
|  | 21_1 | AX-76233198 | 1,313,251 – 1,314,305 | 1.06 | -- |
| GIZZP | 1_65 | AX-75497815 | 65,317,661 – 65,354,646 | 36.99 | *IAPP, PYROXD1* |
|  | 1_81 | AX-75530207 | 81,929,802 – 81,937,618 | 7.82 | -- |
|  | 1_83 | AX-75533562 | 83,599,482 – 83,619,780 | 20.30 | *F5* |
|  | 12_12 | AX-75684414 | 12,178,265 – 12,189,860 | 11.60 | *FHIT* |
|  | 18_4 | AX-75899177 | 4,803,785 – 4,806,988 | 3.20 | *SAP30BP* |
|  | 18_5 | AX-75900424 | -- | -- | -- |
|  | 18_6 | AX-75905027 | 6,476,074 – 6,483,513 | 7.44 | *gga-mir-1561* |
|  | 18_7 | AX-75908553 | 7,469,286 – 7,485,829 | 16.54 | *PRKCA* |
|  | 28_0 | AX-76388399 | 709,716 – 712,969 | 3.25 | -- |
|  | 28_1 | AX-76371869 | 1,314,110 – 1,314,922 | 0.81 | *CELF5* |
|  | 28_2 | AX-76375088 | -- | -- | -- |
| LUNGP | 1_163 | AX-75321597 | 163,850,932 – 163,852,934 | 2.00 | -- |
| INTES | 4_71 | AX-76720974 | 71,293,421 – 71,493,383 | 199.96 | ENSGALG00000030680 |
|  | 4_72 | AX-76723603 | 72,894,460 – 73,027,734 | 133.28 | -- |
|  | 4_73 | AX-76723835 | 73,028,747 – 73,076,028 | 47.28 | -- |
|  | 7_34 | AX-77034722 | 34,021,939 – 34,039,608 | 17.67 | ENSGALG00000041782 |
|  | 7_36 | AX-77040119 | -- | -- | -- |
|  | 15_11 | AX-75819843 | 11,252,960 – 11,292,647 | 39.69 | *DTX1, RASAL1, WSB2, RFC5*, ENSGALG00000036227, *KSR2* |
|  | 19_6 | AX-75937568 | 6,621,916 – 6,639,995 | 18.08 | *P2RX5, EMC6* |
|  | 27_3 | AX-76362680 | 3,648,900 – 3,651,312 | 2.41 | *IGF2BP1* |

LIVP: liver weight as a percentage of body weight; GIZZWT: gizzard weight; GIZZP: gizzard weight as a percentage; LUNGP: lung weight as a percentage; INTES: intestine length.

^1^ Map position based on *Gallus_gallus*-5.0 assembly (NCBI).

^2^ Ensembl gene name and ID based on Galgal5 (*Ensembl Genes 93 Database*).
